# Non-randomised feasibility study of training workshops for Talking Therapies service high-intensity therapists to optimise depression and anxiety outcomes for individuals with co-morbid personality difficulties: a study protocol

**DOI:** 10.1186/s40814-023-01394-z

**Published:** 2023-10-05

**Authors:** Laura A. Warbrick, Barnaby D. Dunn, Paul A. Moran, John Campbell, David Kessler, Katie Marchant, Michelle Farr, Mary Ryan, Megan Parkin, Richard Sharpe, Katrina Turner, Mona Sylianou, Gemma Sumner, Emma Wood

**Affiliations:** 1 Mood Disorders Centre, University of Exeter, Exeter, EX4 4QQ UK; 2 College of Medicine and Health, University of Exeter, Exeter, UK; 3 Bristol Medical School, University of Bristol, Bristol, UK; 4 The National Institute for Health and Care Research Applied Research Collaboration West (NIHR ARC West) at University Hospitals Bristol and Weston NHS Foundation Trust, Bristol, UK; 5 Royal Devon University Healthcare NHS Foundation Trust, Tiverton, UK; 6 Public Health Cornwall, Truro, UK; 7 Everyturn Mental Health, Newcastle Upon Tyne, UK

**Keywords:** Personality difficulties, NHS Talking Therapies, IAPT, Therapist training, Feasibility study, Mixed methods, Cognitive behavioural therapy

## Abstract

**Background:**

The NHS Talking Therapies for Anxiety and Depression programme (‘TTad’; formerly Improving Access to Psychological Therapies ‘IAPT’) delivers high-intensity cognitive behavioural therapy (CBT) to over 200,000 individuals each year for common mental health problems like depression and anxiety. More than half of these individuals experience comorbid personality difficulties, who show poorer treatment outcomes. TTad therapists report feeling unskilled to work with clients with personality difficulties, and enhancing the training of TTad therapists may lead to improved treatment outcomes for individuals presenting with secondary personality difficulties alongside depression and anxiety.

**Methods:**

This is a pre-post non-randomised mixed-method feasibility study, exploring the feasibility and acceptability of a 1-day training workshop for high-intensity (HI) CBT therapists. The workshop is focused on understanding and assessing personality difficulties and adapting HICBT treatments for anxiety and depression to accommodate client needs. The feasibility and acceptability of the workshop and the evaluation procedures will be investigated. It will be examined to what extent the workshop provision leads to improvements in therapist skills and confidence and explored to what extent the training has the potential to enhance clinical outcomes for this client group.

**Discussion:**

This feasibility study will provide data on the acceptability and feasibility of delivering brief therapist training to adapt usual HICBT to optimise care for individuals with secondary personality difficulties seeking treatment in TTad services for a primary problem of depression and/or anxiety. The study will also evaluate proof of concept that such an approach has the potential to improve clinical outcomes for those with secondary personality difficulties and report any possible harms identified. The study will inform the design of a future randomised controlled trial designed to test the effectiveness and cost-effectiveness of the training.

**Trial registration:**

ISRCTN81104604. Submitted on 6th June 2022. Registration date: 3rd January 2023.

**Supplementary Information:**

The online version contains supplementary material available at 10.1186/s40814-023-01394-z.

## Background

NHS Talking Therapy for Anxiety and Depression (TTad) provides access to evidence-based psychological therapies for common mental health problems (predominantly anxiety and depression) in primary care across the English National Health Service (NHS). Formerly known as Improving Access to Psychological Therapy (IAPT) services, in 2021, these services received 1.81 million referrals for talking therapies, with over 660,000 going on to complete a course of treatment [1]. TTad services span Steps 2 or ‘low-intensity (LI)’ and 3 or ‘high-intensity (HI) treatments of the stepped-care model of mental health service delivery) [2]. In 2021, the most commonly delivered treatment was a HI cognitive behavioural therapy (CBT)—accounting for 46% of courses, followed by a LI format—book-assisted guided self-help (26.4%) [1].

Many individuals accessing TTad treatments experience clinically significant improvement (66.9% reliably improved in 2020–2021) and move into recovery (50.2% recovered in 2020–2021) [1]. However, this leaves almost a third who do not show clinically significant improvement and almost half of those who are treated who do not recover. One way to improve treatment outcomes is to identify subgroups of individuals who are not currently showing optimal outcomes and to refine the treatment they are offered to better meet their needs. It is increasingly clear that current difficulties managing emotions, relationships, and sense of self (often associated with exposure to early life adversity or trauma) are linked to poorer response to standard TTad treatments for depression and anxiety [3–5]. These presenting issues are sometimes referred to as ‘personality difficulties’ or ‘complex emotional difficulties’ and are believed to fall on the milder end of the personality disorder spectrum (see the dimensional personality disorder framework proposed in ICD-11) [6–8]. While not routinely screened for, pilot initiatives have demonstrated that between 69 to 81% of TTad clients present with emotional, interpersonal, and identity difficulties associated with ‘personality difficulties’ [5, 9].

While these individuals with personality difficulties do still benefit from TTad interventions, their rates of clinically significant improvement and recovery are lower than in those individuals without personality difficulties. For example, recovery rates for this group are estimated to be around 40%, below the 50% national target, and the 50.2% observed recovery rate observed across all client groups in 2020–2021 [1, 10]. These effects held over and above intake depression and anxiety severity, other demographic features, and number of treatment sessions attended [4, 5].

A subtly different pattern emerges in reviews of CBT outcomes in the clinical trials literature [11, 12]. While there is evidence that clients with a comorbid personality disorder diagnosis have higher symptom levels at the end of CBT treatments for both anxiety and depression, this effect does not consistently emerge when controlling for intake depression and anxiety symptom severity [11, 12]. These secondary analyses are at risk of type II error as the studies were not powered for subgroup analyses and did not stratify clients to treatment on the basis of personality disorder subgroup status. Nevertheless, current data suggest that therapists delivering CBT under ideal conditions can achieve broadly comparable levels of change in clients with and without a comorbid diagnosis of personality disorder. This suggests therapists in TTad settings may benefit from additional training to optimise their ability to deliver existing CBT treatments in the context of these more complex presentations, rather than there being a need to introduce entirely new treatment protocols.

In qualitative evaluations, TTad clients with personality difficulties describe how care has not always felt tailored to their needs [13] and TTad clinicians report feeling unskilled and lacking in confidence to undertake therapy with this client group, and propose core TTad training should include focused training on this topic [14]. In particular, clients viewed more structured approaches as less acceptable and valued more flexible approaches [13], and therapists were less likely to report difficulties ‘maintaining control’ over sessions if they adopted more flexible approaches [14]. Furthermore, recent updates to clinical guidance on depression now reflect the importance of assessment of interpersonal difficulties and tailoring care to cover these issues where they are acting to maintain depression [15].

Despite increasing awareness and adoption of a dimensional framework, and clinical guidance now stating that individuals with this diagnosis should not be routinely denied access to treatments for depression/anxiety [15], ‘personality disorder’ continues to be poorly understood, stigmatised [16], and be associated with inequitable access to care [17]. Previously, the use of targeted educational interventions like the co-produced Knowledge and Understanding Framework [18] and e-learning packages have been shown to lead to significant shifts in mental health workforce attitudes towards personality disorders and workforce burnout in other health care settings [19–22]. Broader literature also shows mental illness stigma can be significantly improved by interventions that shift categorical to continuum beliefs [23].

In sum, concurrent personality difficulties appear to predict poorer outcomes in TTad services, which may be distinct from outcomes in research settings. Outcomes in the TTad setting may be improved by targeting therapist attitudes and understanding of a dimensional framework of personality difficulties and enhancing their clinical skills to assess these difficulties (including whether they can be appropriately managed in a TTad setting) and tailor depression and anxiety treatments accordingly. One significant opportunity to upskill existing workforces with minimal costs involved is to use existing continuing professional development (CPD) time to deliver focused clinical workshops, which may translate into improved clinical outcomes for clients, as well as potentially increasing therapist job satisfaction and wellbeing. This pragmatic approach to improving clinical outcomes for clients with concurrent personality difficulties in TTad services has not previously been evaluated.

This observational study aims to explore the preliminary feasibility and acceptability of a 1-day workshop focusing on enhancing the knowledge, skills, and confidence of HICBT therapists to assess and make evidence-based adaptations to depression and anxiety treatments for TTad clients with concurrent personality difficulties. The study will also examine if the workshop has any impact on workforce wellbeing and assess preliminary proof of concept that training has the potential to lead to improvements in clinical outcomes for clients with co-morbid personality difficulties accessing HICBT treatments for depression and anxiety treatments.

## Methods/design

### Objectives

The over-arching aim of this study is to evaluate the feasibility and acceptability of the training intervention.

The secondary aims of the research are to (1) establish proof of concept that training leads to positive changes in therapist attitudes towards working with this group and improved confidence in key skills covered in training, (2) evaluate the impact of training on therapist wellbeing and burnout, and (3) evaluate preliminary proof of concept that the training has potential to improve outcomes for individuals with co-morbid personality difficulties.

We will set and evaluate continuation rules to inform continuation to definitive randomised evaluation.

### Design

We will conduct a pre-post non-randomised mixed-methods feasibility study, comparing therapist attitudes and workplace wellbeing from before and after the training workshop. We will also conduct a preliminarily pre-post evaluation of routinely collected service-level clinical outcomes on clients attending for treatment that meet the criteria for personality difficulties.

### Study setting

We will purposefully recruit at least three TTad services, chosen to reflect diversity in terms of urban/rural context, levels of deprivation, and ethnicity. Services willing to screen for personality difficulties as part of routine assessment will be eligible to participate as a research site.

### Participants

All HICBT therapists employed by participating TTad services will be invited to attend the training and participate in the research.

### The intervention

The 1-day workshop has three main themes: (1) psychoeducation about personality difficulties; (2) building skills, knowledge, and motivation to better meet the needs of clients with personality difficulties when delivering a CBT protocol for anxiety or depression; and (3) highlighting the importance of, and considering how to build, therapist self-care and resilience when working with more complex clients. The content of the workshop is informed by the CBT evidence base, including guidance about how to adapt CBT for complex cases [24, 25] and for personality disorders [26, 27]. The intention is to make ‘explicit’ what is often ‘implicit’ good practice in how TTad therapists are supporting this client group in a way that supports fidelity to the CBT treatment protocol rather than leading to unhelpful therapist drift.

The training is delivered by two experienced CBT trainers (online or face-to-face) and is a combination of didactic teaching, small group exercises, role play illustration, and role play practice of the techniques; in line with the declarative-procedural-reflective (DPR) model of therapist knowledge acquisition and the COM-B model of behaviour change [28, 29]. A detailed logic model of change underpinning the training (developed retrospectively) is included in Additional file 1: Appendix 1.

The objectives of theme 1 are to build therapist knowledge and understanding about the dimensional framework for personality disorders proposed in ICD-11 [7, 8, 30]; to better understand how these features relate to therapy outcomes; to reduce stigma and ambivalence to working with these clients; and to build skills to assess current severity accordingly of these features to inform if a client is likely to benefit from therapy in a TTad service. Updates to clinical guidance are also covered, highlighting that individuals with comorbid formal personality disorder diagnoses should not routinely be denied access to treatments for depression/anxiety [15], although those with more severe presentations may be more appropriately managed in secondary care services like community mental health teams or specialist personality disorder teams rather than in primary care TTad settings.

Theme 2 aims to build therapist knowledge, skill, and motivation (including confidence) to deliver CBT to clients with personality difficulties alongside their depression/anxiety in a way that meets their needs but remains adherent to core CBT principles. The ground covered draws on both guidance and research evidence and includes managing the therapeutic alliance in clients who may have an ambivalent relationship to help (c.f. [24, 31–33]); how to structure sessions with clients who may present in a ‘stably unstable’ fashion [27]; how to formulate interpersonal difficulties in CBT terms [26, 27, 33]; how to help clients build skills in managing emotions and being interpersonally effective (c.f. [34–40]); how to help clients notice strengths and moments of resilience; and how to manage ruptures and risk in sessions (e.g. [26, 27]). The focus is on supporting therapists to ‘do CBT basics well’ in a way that would result in high scores in competence assessment of a depression or anxiety protocol.

Theme 3 runs throughout the workshop, aiming to validate the therapist’s experience of working with complex clients in a pressured TTad context and supporting therapists to manage their own self-care and resilience.

The workshop has been developed by BD from teaching originally delivered as part of core clinical training for HICBT therapists, aiming to support therapists to transition from ‘university’ practice to ‘real world’ practice by teaching key skills in finding flexibility within treatment protocols while maintaining fidelity to the model to meet the needs of more complex TTad clients. Due to the demand from TTad services for training workshops focused on personality difficulties, this training intervention was developed with this specific client group in mind. Refinement of the training over time has included drawing upon feedback from co-facilitators (experienced CBT therapists and trainers), delegate feedback, updates to clinical guidance (e.g. [8, 15]) and research findings (e.g. [4, 5, 9, 11, 13, 14, 34–40]), and expertise from individuals with lived experience and other stakeholders, including clinicians and service leads. The current version of the workshop has been delivered as a routine CPD workshop in three large TTad services, and a single-site pilot study is currently underway. However, an adequately powered evaluation of therapist outcomes such as the present protocol describes has not yet been undertaken.

### Outcomes

We will collect a range of therapist and clinical outcome measurements to address our primary and secondary research aims (See Table 1). Our primary acceptability and feasibility outcomes relating to methodological and procedural uncertainties are described in Table 2, alongside continuation rules to be met in order to progress to definitive trial evaluation. If these rules are not met, it will be assessed whether the project is still viable if modifications are made or whether to stop the project.

**Table 1:** Therapist participant and secondary clinical data outcome measurements by time point

Outcome	Measure	Time point
**Therapist participant outcomes**		
** Demographics**	Gender, ethnicity, age group, experience level	Pre-training
** Attitudes**	Bespoke attitudinal questionnaire capturing therapist-perceived confidence and competence in working with clients with personality difficulties (for example, ‘I feel confident recognising, assessing, and deciding whether to take on clients with personality difficulties’) on 5 items (see Additional file 2: Appendix 2). Participants will be asked to judge to what extent the statement is true of them on a 5-point Likert scale from strongly disagree to strongly agree. There are no standard measures fit for purpose (to capture attitude and confidence towards working with individuals with personality difficulties). This novel measure has been piloted in previous workshops for TTad HICBT therapists.	Pre-training; Post-training; 3-month follow-up
** Quantitative workshop feedback**	Therapists will answer a series of 4 questions about the delivery and content of the workshop (for example, ‘I found the workshop theoretically interesting’), rated on a 5-point Likert scale from ‘Strongly disagree’ to ‘Strongly agree’ (Additional file 3: Appendix 3). This 4-item questionnaire captures perceived theoretical interest, usefulness and presentation quality and acceptability of the training; and whether they would recommend to other therapists. There is no standard measure fit for this purpose; however, this bespoke scale has been piloted to capture feedback on previous workshops for TTad HICBT therapists.	Post-training
** Qualitative workshop feedback**	Written qualitative questions capturing therapist feedback on training	Post-training
** Wellbeing**	Therapist wellbeing will be assessed using the (adjusted) Short Warwick-Edinburgh Mental Wellbeing Scale (SWEMWBS) [41] 7-item (self-report measure capturing positive wellbeing experiences (for example, ‘I’ve been feeling optimistic about the future’). Therapists will rate the frequency of positive experiences on a 5-point Likert scale from ‘None of the time’ to ‘All of the time’. This version of the scale is adjusted to capture experiences over the past two weeks (adjusted from past week). This measure has previously been used to assess workforce wellbeing [42].	Pre-training; 3-month follow-up
** Impact of training**	Written qualitative questions capturing therapist experiences of training impacts	Post-training; 3-month follow-up
** Burnout**	Sussex Burnout Scale (SBS) [43]—a 3-item self-report measure of frequency of symptoms of burnout at work (for example, ‘I have little or no energy at work or feel exhausted by my job’). Participants will rate frequency of symptoms over the past month on a 5-point Likert scale from ‘Rarely/Never’ to ‘Everyday/Almost every day’ over the past month.	Pre-training; 3-month follow-up
** Presenteeism and absenteeism**	Therapist presenteeism/absenteeism—presenteeism and absenteeism questionnaire (Additional file 4: Appendix 4)—4-item self-report measure capturing frequency of presenteeism and absenteeism from work (for example, ‘At work I have been bothered by physical or psychological problems’). Participants will be asked to rate the frequency of these experiences over the past month from ‘Not at all’ to ‘Nearly every day’. This novel measure will be used as there are no standard measures available fit for the purpose (brief, self-reported measure).	Pre-training; 3-month follow-up
**Routine clinical outcomes on clients with personality difficulties (measured in 6 m before and after the workshop)**		
** Depression symptoms**	Patient Health Questionnaire (PHQ-9 [44];)—a 9-item self-report measure of frequency of depression symptoms (for example, ‘Little interest or pleasure in doing things’). Respondents are asked to rate frequency of symptoms over the past 2 weeks on a 4-point Likert scale from ‘Not at all’ to ‘Nearly every day’. This scale is validated for use in adolescents and adults.	First and last session
** Anxiety symptoms**	The Generalised Anxiety Disorder scale (GAD-7; [45])—a 7-item measure of anxiety symptom (for example, ‘Feeling nervous, anxious or on edge’) frequency over the past 2 weeks, scored on a 4-point Likert scale from ‘Not at all’ to ‘Nearly every day’.	First and last session
** Work and social functioning**	Work and Social Adjustment Scale (WSAS; [46])—a 5-item measure of impairment in daily functioning (for example, ‘Because of my [problem] me ability to work is impaired’) rated on a 9-point Likert scale from ‘Not at all’ to ‘Very severely’.	First and last session
** Measure of personality difficulties**	Standardised Assessment of Personality: Abbreviated Scale—Self Report version (SAPAS-SR; [47, 48])—8-item binary response (yes/no) measure of personality difficulties (for example, ‘In general, do you have difficulty making and keeping friends. This scale is adapted for use as a self-report questionnaire and respondents are asked to indicate ‘Yes’ when the experience applies to them ‘most of the time in most situations’. This scale has been widely used for rapid screening for personality difficulties within the TTad setting for research purposes and has been adopted into routine practice in some services [4, 5, 9].	First session /instance
** Qualitative experiences of treatment**	Patient Experience Questionnaires	Treatment end

**Table 2:** Feasibility and acceptability data, method of measurement, and continuation rule to proceed to randomised evaluation without modification

Feasibility/acceptability outcome	Measurement	Continuation rule
**Recruitment**	*Quantitative data*	
	Number of TTad sites recruited to the research project	≥ 3 IAPT sites recruited
	Percentage of eligible therapists attending the training at each site	≥ 60% attendance
	Percentage of attending therapists completing pre- and post-training surveys	≥ 80% data availability
	Percentage of attending therapists completing 3-month follow-up surveys	≥ 60% data availability
**Acceptability**	*Quantitative data*	
	Feedback data on training (that that the training was theoretically interesting, clinically useful, well presented, and would recommend to other HICBT therapists)	60% agree or strongly agree with each item
	*Qualitative data*	
	Written feedback from therapists on the value of training	No significant concerns emerge about training that cannot be resolved
	Qualitative interviews with therapists	No significant concerns emerge about training that cannot be resolved
	Reporting/identification of serious concerns about the acceptability and feasibility of training by key stakeholders	No serious concerns emerge about training that cannot be resolved
	Reporting/identification of serious negative consequences for therapist participants or the clients they subsequently work with as a result of the training (unexpected, clearly research- or training intervention-related serious adverse reactions).	No serious consequences raised
**Clinical outcome data completion**	*Quantitative data*	
	Percentage of TTad clients receiving routine care during the study with sufficient data for inclusion in secondary analyses (at least one measure of SAPAS-SR personality difficulties and at least × 2 measures of the TTad service minimum dataset [PHQ-9, GAD-7 and WSAS])	≥ 60% data availability
**Proof-of-concept**	*Quantitative data*	
	Therapist attitudinal change in confidence to recognise, assess and triage clients with personality difficulties; to adapt key skills for working therapeutically with this group; and in positivity towards working with this group after completing the training in each service.	Significant improvement from pre to post in all 5 attitudinal domains

In addition to these measures, we will capture clinical data to enable us to generate metrics of engagement including discharge code and number of sessions marked ‘Attended’, ‘Did Not Attend’, and ‘Patient Cancelled’. In line with standard reporting of TTad outcomes, we will also compute the following metrics from clinical outcomes: (i) ‘Recovery’—as defined by moving ≥ 10 to ≤ 9 on the PHQ-9 and from ≥ 8 to ≤ 7 on the GAD-7; (ii) ‘Reliable Improvement’—as defined by improving by ≥ 6 points on the PHQ-9 or ≥ 4 points on the GAD-7; and (iii) ‘Reliable Recovery’—as defined by fulfilling criteria for both ‘recovery’ and ‘reliable improvement’ [49].

### Participant timeline

Pre-training surveys will be distributed 2 weeks before the training workshop. Post-training surveys will be distributed at the end of the workshop and follow-up surveys 3 months after the training. All HICBT therapists employed at participating sites will have the opportunity to attend the training, regardless of their intention to participate in the research. All training attendees will be invited to take part in the research.

### Qualitative interviews

Approximately 1 month after the training, a subsample of therapists who took part in the training and consented to take part in the research will be invited to take part in a remote (telephone or videoconferencing) qualitative interview to explore their experiences (including any challenges) of treating individuals with personality difficulties in TTad services, their perceptions of the workshop, and whether the workshop has led to any changes in their practice or their workplace wellbeing. We will purposefully sample participants who vary in relation to (i) study site, (ii) quantitative and qualitative written feedback on the workshop, (iii) attitudinal change (pre-post), and (iv) therapist experience level.

The therapist sampled will be contacted by email, provided with a 'Participant Information Sheet’ and given an opportunity to discuss participating with a member of the research team. To ensure consistency across the interviews, a topic guide will be used (Additional file 5: Appendix 5). It will be based on the aims of the research and team discussions and informed by the COM-B model of behaviour change and Normalisation Process Theory [29, 50]. With participant consent, all interviews will be audio-recorded and transcribed verbatim. They will be analysed thematically, using the framework analysis approach [51] to help make comparisons within and across the interviews.

### Sample size

#### Therapists

The final therapist sample size will be determined by the number of therapists participating in the training at each of the three participating sites. Based on our previous experience, we anticipate that approximately 40 therapists will attend at each site (120 total). Based on similar studies examining the effects of training for healthcare professionals on attitudes and behaviours (e.g. [52, 53]), we anticipate the effect size of training on change in therapist attitudes and perceived self-efficacy to work with this client group to be at least *d* = 0.4. A G*Power [54] calculation indicates that the required sample size to detect a medium effect of *d* = 0.4 in a paired samples two-tailed *t* test, with 80% power is *n* = 84. Assuming 120 therapists attend the training, with 80% data completion, we will have 96 participants to analyse. This is sufficient power to detect a small-medium (*d* ≥ 0.27) pre-post effect size on these therapist ratings.

A sub-sample of therapist participants will be invited to take part in an in-depth qualitative interview about their experiences of the training. We anticipate undertaking up to 20 interviews, guided by ‘data saturation/information power’ relevant to the study objectives.

#### Service-level secondary data sample

No clients will be recruited for the study. We will perform exploratory secondary analyses on routinely collected and anonymised clinical outcomes data and Patient Experience Questionnaires, focusing on those with personality difficulties. This will be defined as scoring 3 or more on the Standardised Assessment of Personality: Abbreviated Scale—Self Report version (SAPAS-SR) [47, 48]. Considering TTad caseloads and estimates of personality disorders in this setting, we estimate that 40 therapists in each site (120 in total) will treat > 20 clients each in a 6-month period before and the 6-month period after the training, creating an estimated sample size of > 4800 service-users (~ 2400 before and ~ 2400 after). Based on previous studies examining the prevalence of personality difficulties in TTad services, we anticipate between 69 and 81% will meet the criteria for probable personality disorder [5, 9] in both pre- and post-training cohorts. The purpose of this secondary analysis will be to determine preliminary proof of concept that the training has the potential to improve service level outcomes for this population and to inform the power calculation for a subsequent randomised definitive evaluation.

#### Recruitment

UK TTad service sites will be recruited through existing links between the research team and UK IAPT services and through the dissemination of a research proposal via the ‘Northern IAPT Practice Research Network’. HICBT therapists working for participating services will be initially approached via email from a service manager who holds the list of eligible therapists 2 weeks before the training intervention. This email will include a link to a participant information sheet and consent form hosted on a survey platform describing the training intervention and the accompanying research. Therapists will be able to attend the training as part of their routine Continued Professional Development provision through their employment regardless of whether they decide to take part in the accompanying research.

### Statistical methods

#### Primary analyses

Primary analyses will address the acceptability and feasibility aims of the study. We will describe the number of UK TTad services recruited to the study, the number (%) of total eligible therapists identified that attend the training in each site, and the number (%) of attending therapists that complete pre-post follow-up surveys. To determine post-intervention views on acceptability, participants’ mean (SD) ratings of intervention theoretical interest, clinical utility, whether it was well presented, and whether they would recommend to other HICBT therapists were rated on a 5-point Likert scale (See Table 1).

We will report the number (%) of cases included in the secondary data extraction with sufficient data for inclusion within the clinical outcome evaluation.

#### Secondary analyses

Secondary analyses of therapist outcomes will focus on both therapist outcomes, including proof-of-concept in attitudinal change, wellbeing, and burnout measures and preliminary proof-of-concept that training has the potential to lead to improvements in clinical outcomes for those with personality difficulties.

#### Therapist outcomes

We will report descriptive statistics (means, standard deviations) to describe the attitudes of therapists before and after the training intervention on each individual rating.

Considering pre to post as our primary outcome for therapist attitudinal change, we will use a series of paired samples two-tailed *t* tests (or an equivalent non-parametric test if that data is not adequately normally distributed) and report effect size (Cohen’s D) to establish preliminary proof of concept that training is significantly associated with positive change in therapist reported confidence to recognise, assess, and triage clients with personality difficulties; formulate clients with these difficulties; anticipate challenges to alliance; and adapt key skills for working therapeutically with these clients, in addition to positive change in attitudes towards working with this group. As a secondary exploratory analysis, we will perform repeated measures ANOVAs including all three timepoints (pre-, post-, and 3-month follow-up).

If we recruit a sufficient sample size (at least 120 × therapists across the 3 sites, with 80% data completion, and therefore 96 complete cases to analyse), we will also use exploratory moderation analyses to explore whether the site, therapist experience (number of years post-qualification), ethnicity (white British vs. other), gender (male/female/non-binary), and experience level moderate the extent of change in attitudes observed during the training.

We will also address whether training has an impact on the rapist-reported wellbeing (SWEMWBS) or workplace burnout (SBI). We will report descriptive statistics to describe the wellbeing and burnout of therapists before and after the training intervention and use paired sample two-tailed tests (or a non-parametric equivalent if data is not adequately normally distributed) to establish if there is significant change in these secondary outcome measures.

#### Clinical outcomes

We will report descriptive statistics (means, standard deviations) to describe the clinical measures and client characteristics at intake within the pre- and post-training intervention cohorts (those engaging in treatment in the 6 months pre- compared to 6 months post-therapist training). This will include PHQ-9, GAD-7, and WSAS mean scores; the proportion of clients showing mild, moderate, and severe cut-offs on each scale; and the proportion of clients scoring 3 or more on the SAPAS-SR (indicative of personality difficulties).

This research is a multi-site observational study prior to conducting a definitive evaluation with a randomised trial and therefore has not been a priori powered to inferentially examine between cohort differences in clinical outcomes. However, it is both possible and useful to estimate the between-cohort effect size (and their 95% confidence interval) on client outcomes to help inform decisions to continue to a definitive evaluation of the training intervention and to inform future power calculations. We will therefore report the observed effect sizes but not *p* values. These analyses will focus solely on the subgroup of participants meeting the criteria for probable personality disorder (3 or more on the SAPAS-SR), which we estimate from previous research to be between 69 and 81% of client treatments [5, 9]. A series of linear regressions will examine whether treatment-cohort (treated before or after training) predicts the number of treatment sessions attended and post-treatment levels of depression (PHQ-9), anxiety (GAD-7), and functioning (WSAS). All analyses will include the training site as a covariate. The post-treatment analyses will adjust for the pre-treatment level of the relevant variable. Comparable binary logistic regressions will examine binary outcomes (rates of reliable improvement, recovery, and reliable recovery; rates of clients with planned discharges), again seeing if the treatment cohort (before or after training) predicts each variable and covarying for the training site. As we do not anticipate sufficient statistical power to do inferential statistics, we will examine the distribution of effect sizes, potentially using confidence intervals and Bayesian methods relative to estimates of minimum clinically important difference on clinical outcomes (cf., [55–58]). All quantitative analyses will take place in SPSS or R.

#### Qualitative analysis

Written qualitative feedback will be anonymised and interviews transcribed verbatim and anonymised prior to any analysis. Both datasets will be explored using a framework method to support the systematic refinement of themes to understand experiences and their meanings [30, 51]. Analysis of therapist qualitative data will be both inductive and deductive, informed by behavioural change theory (COM-B [29] and Normalisation Process Theory [50]) in order to explore barriers and enablers to implementation of the skills practiced during the training, as well as the implementation of this training workshop more broadly into routine HICBT therapist training. The analysis will be an iterative process involving close reading and familiarisation with the data, coding, comparison, and refinement and elaboration of emerging themes. NVivo will be used to support qualitative data management.

Findings will indicate therapists’ views on the feasibility and acceptability of the training and the research procedures involved. They will inform future refinement of the training intervention, as well as whether trial procedures need amendment before continuation to a definitive trial.

## Discussion

Individuals with comorbid personality difficulties make up a significant proportion of the TTad population and have a poorer response to depression and anxiety treatments compared with those without these additional difficulties [4, 5, 9]. TTad clinicians also report feeling unskilled to undertake this work, despite recognising that working with this population is central to their care context [13, 14]. Therefore, delivering additional training to TTad clinicians represents a significant opportunity to improve therapist knowledge, skill, and confidence and to improve care and outcomes for clients.

Other research has reported that training interventions for healthcare staff to improve understanding of personality disorders has led to shifts in workforce attitudes towards individuals with personality disorders and reductions in staff burnout (e.g. [18, 19]). However, interventions tailored to a HICBT workforce aiming to enhance skills specific to their type of clinical work have not previously been formally evaluated. This study will therefore, for the first time, evaluate a HICBT therapist-specific training workshop across multiple TTad services to improve understanding and tailoring of care for individuals with secondary personality difficulties. However, the current training focuses solely on high-intensity therapists, which is only a subset of the TTad workforce. In due course, we think there is a need to develop and evaluate comparable training for the low-intensity (Psychological Wellbeing Practitioner) therapists, who deliver a large proportion of care and often conduct initial intake assessments in a majority of TTad services.

Through an embedded secondary analysis of routine clinical outcomes, this study will also establish whether training shows the potential to lead to improvements in clinical outcomes for individuals receiving TTad treatments who present with secondary personality difficulties.

Together these findings will inform the future investigation of this approach and whether to proceed to a definitive randomised controlled trial powered to test the clinical effectiveness and cost-effectiveness of the training intervention.

### Study status

Participant recruitment will begin in March 2023.

### Supplementary Information

**Additional file 1: Appendix 1.** Detailed logic model of change underpinning the training workshop.

**Additional file 2: Appendix 2.** Bespoke attitudinal questionnaire.

**Additional file 3: Appendix 3.** Bespoke workshop feedback questionnaire.

**Additional file 4: Appendix 4.** Presenteeism/absenteeism questionnaire.

**Additional file 5: Appendix 5.** Therapist Interview topic guide. Developed by Michelle Farr.

## Data Availability

Study documentation including full study protocol, data collection survey templates, and intervention materials can be requested from the corresponding author. At the point of submission of the manuscript, no data has been collected or analysed as part of this study.

## Abbreviations

CBT: Cognitive behavioural therapy
DPR: Declarative-procedural-reflective
GAD-7: Generalised Anxiety Disorder 7 questionnaire
HI: High intensity
IAPT: Improving Access to Psychological Therapies
LI: Low intensity
NHS: National Health Service
PHQ-9: Patient Health Questionnaire 9 questionnaire
SAPAS-SR: Standardised Assessment of Personality: Abbreviated Scale – Self Report version
SBS: Sussex Burnout Scale
SWEMWBS: Short Warwick-Edinburgh Mental Wellbeing Scale
TTad: NHS Talking Therapy for Anxiety and Depression
WSAS: Work and Social Adjustment Scale
