# Supplementary material for: Non-randomised feasibility study of training workshops for Talking Therapies service high-intensity therapists to optimise depression and anxiety outcomes for individuals with co-morbid personality difficulties: a study protocol
Source: Pilot Feasibility Stud. 2023 Oct 5;9:170. doi: 10.1186/s40814-023-01394-z (PMC10552316; doi:10.1186/s40814-023-01394-z)
Supplement: Supplementary file 1 — Additional file 1: Appendix 1. Detailed logic model of change underpinning the training workshop. [file 40814_2023_1394_MOESM1_ESM.pdf]

Problem Model

Capability

- Lack of knowledge and understanding of personality difficulties and disorder
- Lack of knowledge and understanding of impact on personality difficulties/disorder on depression and anxiety
- Lack of knowledge and skills to normalise experiences of personality difficulties/disorder
- Lack of knowledge and skills in assessing for personality difficulties and disorder
- Lack of knowledge and skills in differentiating between those suitable for treatment within IAPT, and those to be referred/signposted on.
- Lack of knowledge and skills to discuss personality difficulties/disoder
- Lack of knowledge and skills around building client positive relationship to help
- Lack of knowledge and skills in managing challenging therapeutic alliance
- Lack of knowledge and skills in managing end of therapy
- Lack of knowledge and skills in supporting client to be environmental change agents around relationships, work and other parts of NHS system
- Lack of knowledge/skills in building client emotion regulations skills
- Lack of knowledge/skills in formulating co-occuring personality difficulties/disorder
- Lack of knowledge/skills in adapting usual practice to cope with cooccuring personality difficulties/disorder
- Lack of knoweldge/skills in building client interpersonal effectiveness
- lack of knowledge and skills in boundary setting during therapy
- Lack of knowledge and skills in managing ruptures during therapy
- Lack of knowledge and skills in managing crises during therapy
- Lack of skills to maximise use of supervision
- Lack of self-care skills

Motivation

- Low percieved self efficacy to support clients with co-occuring personality difficulties/disorder
- Low self-efficacy in managing ruptures and crises during therapy
- Percieved negative consequences for brief treatments for clients with personality difficulties
- Belief that co-occuring personality difficulty is outside IAPT remit
- Negative believes about clients capacity to engage with therapy
- Negative beliefs about clients capacity to benefit from IAPT treatments
- Perfectionist personal standards regarding therapy delivery
- reluctance to become learner again for experienced therapists
- Negative beliefs about individuals with personality difficulties
- Belief that working with clients with personality difficulties is challenging and not enjoyable
- Some degree of 'burnout' due to ongoing workload pressures and change initiatives

Opportunity

- Lack of clear guidance around suitability for IAPT
- Lack of supportive structure to enable effective crisis management
- Insufficient time to attend training, due to absence of CPD time
- Insufficient time and headspace to reflect on practice during therapy due to high caseload and with back-to-back sessions
- Insufficient time to read materials whilst applying therapy, due to lack of CPD time
- Insufficient regular access to high quality supervision
- lack of peer group to discuss therapeutic work with
- Conflicting/confusing service guidelines/politics around label 'personality disorders'
- Service percieves personality difficulties/disorders as "not IAPT business"
- Working culture holds negative beliefs about individuals with personality difficulties

Therapist Behaviours

Style:

• Over focus on the difficult
• Fails to validate client emotional experience and distress
• Fails to empathise with client experience and distress
• Unable to build and sustain therapeutic alliance (particularly with challenging clients)
• Therapist does not share responsibility for change with client (taking on too much or little responsibility)
• Inconsistent in approach to therapy sessions
• Therapy follows a formal, serious style and is not fun
• Therapy is too protocol driven and formulaic (and not personalied to client needs)
• Over focus on risk management
• Failing to maintain appropriate therapy boundaries (timing, attendance etc)
• Therapy is over-complicated in response to client presenting complex problems
• Fails to recognise and consider personality difficulties within formulation and treatment
• Fails to anticipate working alliance challenges and ruptures
• Therapist fails to attend to own emotional reaction during therapy

Structure:

• Fails to adequately socialise client to CBT structure early on in treatment
•Fails to co-set and follow a realistic agenda
• Fails to make appropriate use of psychoeducation
• Does not encourage client consolidation of learning
•Fails to manage time appropriately in sessions
•Does not monitor outcomes and use this in a way to refine therapy
•Does not seek and act on feedback from client, including around goals, tasks and process
•Fails to have a clear set of objectives for therapy to work towards; is not following a consistent blueprint for change
• Fails to manage boundaries around therapy (missed sessions, late appointments etc.), prematurely ends therapy
• Fails to adapt structure according to client moodstate

Specific processes

• Does not help client build key skills in emotional regulation and interpersonal effectiveness
• Does not address client relationship to help or foster positive relationship to help
• Does not adequately target interventions that build client self-efficacy
• Does not help client deal adaptively with aversive emotions
• Does not focus on building behavioural competence and assertiveness skills
•Does not foster trusting relationship, support and safety and help clients evaluate threat
• Does not engage with positive aspects of client and areas of life going well

Therapist Learning Style

In session:

•Does not experiment with novel techniques/approaches in session
•Wishes to rigidly follow protocol or alternative to go off piste
•Does not monitor outcomes and use this in a way to refine own practice
•Does not prepare fully for sessions
•Therapist does not listen to own tapes or complete fidelity ratings scales

Supervision and study:

• Does not regularly attend supervision
• Does not prepare fully for supervision
• Therapist is not fully engaged in supervision, including willingness to engage in role play, discuss process, and to experiment with novel techniques/approaches in supervision
• Therapist engages in a problem saturated rather than solution focused dialogue in supervision
• Therapist does not seek client, peer or supervisor feedback
• Therapist is defensive in face of feedback
• Therapist does not reflect on and refine practice, including committing to behavioural experiments in session and in own life based on supervision feedback
• Therapist does not provide constructive feedback to peers
•Therapist does not consolidate own learning (e.g. note-taking, diary keeping)
• Therapist does not appropriately liaise with other parts of the health system

Broader Life:

•Therapist does not engage in self-care

Clinical Outcomes

- Client is not fully engaged in treatment
- Treatment less effective
- Key aspects of clients lives are not worked on
- Higher subsequent levels of relapse
- Therapist does not enjoy therapy or feel they are doing a good job
- Client does not develop adaptive relationship to help
- Client does not enjoy therapy or feel liked by therapist

Capability

-Have knowledge and understanding of personality difficulties and disorder
- Have knowledge and understanding of impact on personality difficulties/disorder on depression and anxiety
- Have knowledge and skills to normalise experiences of personality difficulties/disorder
-Have knowledge and skills to assess for personality difficulties and disorders
-Have knowledge and skills in differentiating between those suitable for treatment within IAPT, and those to be referred/signposted on
- Have knowledge and skills to discuss personality difficulties/disorder with clients
- Have knowledge and skills around building client positive relationship to help
- Have knowledge and skills in managing challenging therapeutic alliance
-Have knowledge and skills in managing the end of therapy
-Have knowledge and skills in supporting client to be environmental change agents around relationships, work and other parts of NHS system.
- Have knowledge and skills in building client emotion regulation skills
- Have knowledge and skills in formulating co-occurring personality difficulties/disorder
- Have knowledge and skills in building client interpersonal effectiveness
-Have knowledge and skills in boundary setting during therapy
- Have knowledge and skills in managing ruptures during therapy
- Have knowledge and skills in managing crises during therapy
-Have developed skills to maximise use of effective supervision
-Have developed self care skills

Motivation

- High perceived self efficacy to support clients with co-occurring personality difficulties/disorder
- High perceived self-efficacy in managing ruptures and crises during therapy
- Perceived positive consequences for brief treatments for clients with personality difficulties
- Belief that co-occurring personality difficulty is within IAPT remit
- Realistic but positive believes about clients capacity to engage with therapy
- Realistic but positive beliefs about clients capacity to benefit from IAPT treatments
- Realistic, 'good enough' personal standards regarding therapy delivery
- Willingness to become learner again for experienced therapists
- Realistic but positive beliefs about individuals with personality difficulties
- Belief that working with clients with personality difficulties is within own capabilities and enjoyable
- Manageable workload pressures and change initiatives, meaning therapist is engaged

Opportunity

- Sufficient clear guidance around suitability for IAPT
- Sufficient supportive service structure to enable effective crisis management
- Sufficient time to attend training, due to absence of CPD time
- Sufficient time and headspace to reflect on practice during therapy, due to manageable caseload with breaks between sessions
- Sufficient time to read materials whilst applying therapy, including allocated CPD time
- Sufficient regular access to high quality supervision
- lack of peer group to discuss therapeutic work with
- Consistent clear messaging from service leads around label 'personality disorders' and subthreshold personality difficulties
- Service is inclusive for clients with depression or anxiety and co-occurring personality difficulties and disorders and takes on clients suitable for primary care based treatments and believes they are "IAPTs business"
-Working culture holds realistic but positive beliefs about individuals with personality difficulties

Therapist Behaviours

Style:

•Balanced focus on the difficult and positive aspects of client and areas going well
• Appropriately validates client emotional experience and distress
• Appropriately empathises with client experience and distress
• Able to build and sustain therapeutic alliance (particularly with challenging clients)
• Therapist shares responsibility for change with client
• Therapist employs a consistent approach to therapy sessions
• Therapy follows a fun, creative, playful but validating style
• Therapy follows protocol and principal, but is delivered flexibly to weave into client agenda.
• Therapist responds with simple clear interventions in the face of complexity
• Therapist recognises and considers personality difficulties within formulation and treatment
• Therapist anticipates working alliance challenges and ruptures
• Therapist attends to own emotional reaction during therapy

Structure:

• Therapist socialises client to CBT structure early on in treatment
•co-sets and follows a realistic agenda
•makes appropriate use of psychoeducation
•sets and reviews appropriate homework
•encourages client consolidation of learning
•manages time appropriately in sessions
•monitors outcomes and uses this to refine therapy
•seeks and act on feedback from client, including around goals, tasks and process
•has a clear set of objectives for therapy to work towards; is not following a consistent blueprint for change
•Manages boundaries around therapy (missed sessions, late appointments, etc.) and is quick to set boundaries, but cautious to end therapy.
•Adapts structure according to client mood state

Training-Specific processes

•Helps client build key skills in emotional regulation and interpersonal effectiveness
•Addresses client relationship to help and fosters positive relationship to help
• Targets interventions that build client self-efficacy
•Helps client deal adaptively with aversive emotions
•Focuses on building behavioural competence and assertiveness skills
•Fosters trusting relationship, support and safety to help clients evaluate threat
•Engages with positive aspects of client and areas of life going well

Therapist Learning Style

In session:

•Willing to experiment with novel techniques/approaches in session
•Follows protocol but in a flexible, principal driven way
•Monitors outcomes and use this in a way to refine own practice
•Prepares fully but efficiently for sessions
•Therapist listens to own tapes and completes fidelity ratings scales

Supervision and study:

•Regularly attend supervision
•Prepares fully for supervision
•Therapist is fully engaged in supervision, including willingness to engage in role play, discuss process, and to experiment with novel techniques/approaches in supervision
•Therapist seeks client, peer and supervisor feedback
•Therapist is reflective and responsive in face of feedback
•Therapist reflects on and refines practice, including committing to behavioural experiments in session and in own life based on supervision feedback
•Therapist provides constructive feedback to peers
•Therapist consolidates own learning (e.g. note-taking, diary keeping)
• Therapist appropriately liaises with other parts of the health system

Broader Life:

•Therapist engages in good enough in self-care at home and at work

Clinical Outcomes

- Client is fully engaged in treatment
- Treatment effective at building wellbeing and reducing depression
- Key aspects of clients lives are worked on
- Reduced subsequent levels of relapse
- Therapist enjoys therapy and feels they are doing a good job
- Client develops adaptive relationship to help
- Client enjoys therapy and feels liked by therapist
