# Supplementary material for: Non-randomised feasibility study of training workshops for Talking Therapies service high-intensity therapists to optimise depression and anxiety outcomes for individuals with co-morbid personality difficulties: a study protocol
Source: Pilot Feasibility Stud. 2023 Oct 5;9:170. doi: 10.1186/s40814-023-01394-z (PMC10552316; doi:10.1186/s40814-023-01394-z)
Supplement: Supplementary file 2 — Additional file 2: Appendix 2. Bespoke attitudinal questionnaire. [file 40814_2023_1394_MOESM2_ESM.docx]

**Appendix 2.** Bespoke attitudinal questionnaire.

Please rate the following statements based on your views following the workshop:

|  | Strongly Disagree (1) | Disagree (2) | Neutral (3) | Agree (4) | Strongly Agree (5) |
| --- | --- | --- | --- | --- | --- |
| 1. I feel confident recognising, assessing and deciding whether to take on clients with personality difficulties |  |  |  |  |  |
| 2. I feel confident anticipating relationship to help and managing the therapeutic alliance in clients with personality difficulties |  |  |  |  |  |
| 3. I feel confident about formulating clients with personality difficulties |  |  |  |  |  |
| 4. I feel confident about managing emotional and interpersonal features of clients with personality difficulties |  |  |  |  |  |
| 5. I feel excited/positive about the prospect of working with clients with personality difficulties |  |  |  |  |  |
