# Supplementary material for: Non-randomised feasibility study of training workshops for Talking Therapies service high-intensity therapists to optimise depression and anxiety outcomes for individuals with co-morbid personality difficulties: a study protocol
Source: Pilot Feasibility Stud. 2023 Oct 5;9:170. doi: 10.1186/s40814-023-01394-z (PMC10552316; doi:10.1186/s40814-023-01394-z)
Supplement: Supplementary file 3 — Additional file 3: Appendix 3. Bespoke workshop feedback questionnaire. [file 40814_2023_1394_MOESM3_ESM.docx]

Appendix 3. Bespoke workshop feedback questionnaire.

Please rate the following statements based on your views on the workshop:

|  | Strongly Disagree (1) | Disagree (2) | Neutral (3) | Agree (4) | Strongly Agree (5) |
| --- | --- | --- | --- | --- | --- |
| 1. I found the workshop theoretically interesting. |  |  |  |  |  |
| 2. I found the workshop clinically useful |  |  |  |  |  |
| 3. I found the workshop well presented |  |  |  |  |  |
| 4. I would recommend the workshop to other high-intensity therapists |  |  |  |  |  |

What did you like about the workshop?

________________________________________________________________

________________________________________________________________

What could be improved about the workshop?

________________________________________________________________

________________________________________________________________

What, if anything will you do differently as a result of the workshop?

________________________________________________________________

________________________________________________________________

What are your views on the opportunities and challenges of working with clients with personality difficulties within Talking Therapies services?

________________________________________________________________

________________________________________________________________

How, if at all, do you think your workplace wellbeing might change as a result of the workshop?

________________________________________________________________

________________________________________________________________

Any other comments?

________________________________________________________________

________________________________________________________________
