# Supplementary material for: Non-randomised feasibility study of training workshops for Talking Therapies service high-intensity therapists to optimise depression and anxiety outcomes for individuals with co-morbid personality difficulties: a study protocol
Source: Pilot Feasibility Stud. 2023 Oct 5;9:170. doi: 10.1186/s40814-023-01394-z (PMC10552316; doi:10.1186/s40814-023-01394-z)
Supplement: Supplementary file 4 — Additional file 4: Appendix 4. Presenteeism/absenteeism questionnaire. [file 40814_2023_1394_MOESM4_ESM.docx]

Appendix 4. Presenteeism/absenteeism questionnaire.

Please read each of the following statements and select how often the statement has applied to you in the past month

|  | Rarely/Never (1) | Less than once a month (2) | 1-3 times a month (3) | 1-3 times a week (4) | Everyday/almost everyday (5) | Prefer not to say (6) |
| --- | --- | --- | --- | --- | --- | --- |
| I have little or no energy at work or feel exhausted by my job (1) |  |  |  |  |  |  |
| I feel mentally distanced from my job or feel negative or cynical about my job (2) |  |  |  |  |  |  |
| I am less effective at my job than I could be (3) |  |  |  |  |  |  |
