# Supplementary material for: Non-randomised feasibility study of training workshops for Talking Therapies service high-intensity therapists to optimise depression and anxiety outcomes for individuals with co-morbid personality difficulties: a study protocol
Source: Pilot Feasibility Stud. 2023 Oct 5;9:170. doi: 10.1186/s40814-023-01394-z (PMC10552316; doi:10.1186/s40814-023-01394-z)
Supplement: Supplementary file 5 — Additional file 5: Appendix 5. Therapist Interview topic guide. Developed by Michelle Farr. [file 40814_2023_1394_MOESM5_ESM.docx]

Appendix 5. Therapist Interview topic guide. Developed by Michelle Farr.

1. Could you give me some details about your role. Make sure they cover:
   1. job title, length of time working in service, when qualified, contact with service users
2. Can you tell me a bit about your work as a Talking Therapies CBT therapist?
3. How often do you work with people with concurrent personality difficulties? Was this apparent from the initial assessment/appointment? How do you find out about these difficulties—is it through initial screening tools like SAPAS and/or PDS-ICD-11 or does it become clear through interactions?
4. What is your understanding of personality difficulties and how they affect people?
5. Previous to the training how have these difficulties affected your sessions with these service users?
6. Do you have any concerns about working with this population? Has this changed since you have taken part in the training session?
7. Why did you decide to take part in the training?
8. What did you hope the training would provide?
9. How long ago was it since you did the training? What do you remember most from it?

**Note to interviewer: NPT coherence**

1. What did you learn about different ways of working with people with personality difficulties? What knowledge did you gain from the training?
2. What did you see as the purpose of the training?
3. Are there new tasks and ways of working that you can take on as a result of the training?
4. What did you see as the value and benefits of the training?

**Note to interviewer: NPT cognitive participation**

1. Do you think that working with people with concurrent personality difficulties should be part of your work? Has your attitude or approach towards this group of service users since the training?
2. Have you been able to change your practice as a result of the training? If so, how?
3. Have you spoken with colleagues about how working practices can be changed following the training? Have there been any team reflections on how the training could be used?
4. What actions and processes have you taken on since the training? Have there been any changes to protocols/ ways of working with this group?

**Note to interviewer: NPT collective action**

1. Have you used tools from the training in your work with service users? Can you see any changes in your interactions/rapport with service users? (Prompts)
   1. Have you been able to understand presenting issues more easily?
   2. Have service users been able to share with you any concerns they have had about potential barriers to recovery?
   3. Have you been able to personalise your approach to individuals’ needs?
2. Did the training provide you with the right mix of skills and knowledge to work with service users with personality difficulties? Was anything missing?
3. Do you feel more confident and trust in your skills since having taking part in the training?
4. What support do you need from managers and the organisation to better embed skills and learning from the training into your practice to work more effectively with this client group?
5. Is there anything else that would support you being able to implement this training in practice?

**Note to interviewer: NPT reflexive monitoring**

1. Has your practice changed when working with service users with concurrent personality difficulties since the training?
2. Can you provide examples of the impacts of the training on your practice? Do you think it has had positive outcomes for clients? If so, what sort of evidence have you seen of this? What sort of outcomes seem to have improved?
   1. In the future, we’re exploring the possibility a larger study that incorporates interviewing service users about their experiences. To explore the potential of this, do you think you’d be able to distinguish clients who you think might have benefitted and/ or not benefitted with respect to the training intervention?
3. Do you have sufficient external support and supervision to help with this work?
4. Would you recommend others undertake the training? Why/ why not?
5. Has the team made changes to working practices since the training?
6. Do you have any recommendations for improving the training?
7. Is there anything else that you’d like to add before we finish?

**Many thanks for your time**

**Normalisation process theory**

- **[C] Coherence**: How is the training perceived? Does it make sense to those involved?
- **[CP] Cognitive participation:** Is there ‘buy-in’ from staff for the implementation work?
- **[CA] CollectiveaAction:** What is the work that needs to happen for implementation to occur?
- **[RM] Reflexive Monitoring**: How is the implementation work evaluated by those involved?
